# Gut microbiota and neuromuscular disorders: a comprehensive review

**DOI:** 10.1055/s-0046-1829037

**Published:** 2026-10-06

**Authors:** Christian Messina

**Affiliations:** 1 Azienda Sanitaria Provinciale Catania, Catania, Italy.

**Keywords:** Gastrointestinal Microbiome, Neuromuscular Diseases, Dysbiosis, Muscles, Microbiota Analysis

## Abstract

Emerging evidence highlights a bidirectional interaction between the gut microbiota (GM) and the neuromuscular system, suggesting that microbial composition and metabolic activity may play a pivotal role in the pathophysiology of several neuromuscular diseases (NMDs). This comprehensive review synthesizes current evidence on GM alterations across a broad spectrum of NMDs, emphasizing their potential implications for disease mechanisms and management. Gut microbial shifts have been consistently associated with immune dysregulation, systemic inflammation, and metabolic disturbances that may contribute to neuromuscular dysfunction. Beyond their pathogenetic relevance, these alterations hold diagnostic and therapeutic promise. Probiotic supplementation, dietary modulation, and other microbiota-targeted strategies have shown preliminary benefits in alleviating symptoms and improving metabolic balance in selected NMDs, although existing studies remain limited and heterogeneous. This review offers an integrative perspective, positioning the GM not only as a potential biomarker for diagnosis and disease monitoring but also as a novel therapeutic target. A deeper understanding of the gut–muscle–nerve axis could pave the way for more personalized and mechanism-based approaches in neuromuscular medicine, particularly for disorders where disease-modifying treatments are still lacking. Further well-designed, longitudinal, and mechanistic studies are warranted to elucidate causal relationships and translate microbiome research into clinical application.

## INTRODUCTION

Human health faces growing threats from environmental and lifestyle changes, with neurological and neuromuscular disorders (NMDs) rising worldwide. The gut microbiome (GM)—a complex ecosystem of bacteria, archaea, viruses, and eukaryotes in the gastrointestinal tract—plays a crucial role in host homeostasis. 1 2 The GM contains an estimated gene pool 100 to 150 times larger than the human genome and harbors approximately 1,000 bacterial species and over 7,000 bacterial strains. 1 2 The predominant bacterial phyla include firmicutes (such as *Lactobacillus* , *Eubacterium* , and *Clostridium* ) and bacteroidetes (notably *Bacteroides* and *Prevotella* ), together constituting the majority of the intestinal microbial community. 1 2 Additional phyla contributing to this ecosystem are actinobacteria, proteobacteria, and verrucomicrobia. 1 2

An imbalance in this microbial composition, referred to as gut dysbiosis, has been linked to a variety of pathological conditions. 1 2 3 The GM influences systemic physiology via metabolites, immune modulation, and neuroendocrine signaling, shaping the gut–brain axis and, more recently recognized, the gut–muscle axis. 2 3 4 Age-related dysbiosis correlates with muscle decline and ‘inflammaging’, partly due to reduced butyrate-producing bacteria. 2 3 4 Notably, a reduction in butyrate-producing bacteria—such as *Clostridium* cluster XIVa and *Roseburia* —appears to be a hallmark of the aging microbiome and may contribute to muscle atrophy. 2 3 4

Experimental interventions targeting the GM, such as probiotics and butyrate supplementation, have improved muscle mass and function in preclinical models. 3 4 For example, supplementation with butyrate in aged mice was found to increase lean muscle mass, supporting a causal link between GM-derived metabolites and muscle physiology. 3 4 Emerging evidence also implicates the GM in NMDs like myasthenia gravis (MG) and muscular dystrophies.

While the role of GM has been extensively investigated in neurological disorders with a strong immunological component, such as multiple sclerosis and neurodegenerative diseases, its potential involvement in NMDs of primarily genetic origin has received comparatively less attention. Nevertheless, increasing evidence suggests that GM may influence disease expression even in genetically determined conditions through mechanisms including immune modulation, metabolic signaling, and regulation of systemic inflammation. Therefore, exploring the gut–muscle axis in NMDs may provide novel insights into disease variability and potential therapeutic targets.

This review provides a comprehensive overview of current findings on GM alterations in NMDs, exploring mechanisms, diagnostic and therapeutic potentials, and future research directions. Table 1 summarizes the currently available evidence on GM alterations across different NMDs, highlighting potential mechanisms, gaps in knowledge, and emerging therapeutic perspectives. To complement this overview, Table 2 focuses specifically on the functional consequences of dysbiosis for each condition, summarizing the main mechanistic effects and their potential impact on disease pathophysiology. Together, these tables offer a comprehensive perspective, allowing readers to understand both the compositional shifts in the GM and the ways in which these alterations may contribute to disease progression and symptomatology.

**Table 1:** Summary of gut microbiota alterations across neuromuscular diseases

Disease	Category	Evidence on GM	Proposed mechanisms	Knowledge gaps	Potential therapeutic implications
MG	Autoimmune	Reduced microbial diversity; decreased Actinobacteria, Firmicutes (Clostridium, Eubacterium, Lactobacillus), Verrucomicrobiota; increased Proteobacteria, Bacteroidetes, Thermodesulfobacteriota; oral microbiota shifts (↑ Streptococcus, Rothia; ↓ Neisseria, Haemophilus, Treponema); altered fecal metabolites	Dysbiosis promotes proinflammatory state, Th17 polarization, impaired Treg induction; altered gut–muscle and gut–brain axis; immune activation at NMJ	Small cohort studies; effect of medications, diet, and lifestyle confounding; causal direction unclear	Probiotics, prebiotics, synbiotics; FMT; microbiome-based biomarkers for stratification and therapy
DMD	Genetic	Reduced microbial richness in mdx mice; altered phyla (actinobacteria, proteobacteria, tenericutes, deferribacteres); increased LPS-producing Gram-negatives (vacteroides, deferribacteres); increased Alistipes, Prevotella in muscle/immune tissues	Dysbiosis and impaired intestinal barrier exacerbate muscle inflammation; SCFA and lipid metabolite alterations activate TLR pathways and oxidative stress; gut–muscle axis modulation	Human data limited; role of dystrophin in gut smooth muscle unclear; causality not established	PPARδ agonists ± AMPK inhibitors; FMT; choline, polyamine, taurine metabolism modulation
MD1	Genetic	Altered firmicutes/bacteroidetes ratio; decreased actinobacteria (Corynebacterium, Rothia, DNF00809), Lactobacillus; increased Clostridium CAG-352, lachnospiraceae, prevotella, bacteroidetes, fusobacterium; altered SCFA profiles	Dysbiosis contributes to systemic inflammation, GI symptoms, and pain modulation; microbial shifts may affect CTG repeat-driven pathogenesis	Mechanistic link between microbiota changes and CTG repeats needs confirmation; pediatric vs adult differences	Probiotics, tailored diets to improve gut function and reduce inflammation
FSHD	Genetic	Increased enterobacterales, veillonellales-selenomonadales, selenomonadaceae, sutterellaceae, veillonellaceae; decreased SCFA producers (Alistipes, Butyricimonas, UCG spp.); increased pasteurellaceae	Dysbiosis and altered SCFA metabolism may influence DUX4 expression; intestinal permeability (“leaky gut”) may contribute to systemic immune activation	Human data scarce; functional impact of SCFA shifts and toxins on muscle cells not fully established	Microbiome-targeted modulation could theoretically affect DUX4 regulation and disease severity
Amyotrophic Lateral Sclerosis (ALS)	Neurodegenerative	Altered F/B ratio (conflicting); reduced SCFA producers (Butyricimonas, Faecalibacterium, Blautia, Ruminococcus, Prevotella); increased Escherichia, Klebsiella, Streptococcaceae; altered sphingolipids, bile acids, BMAA; A. muciniphila ↑ nicotinamide	Dysbiosis promotes intestinal barrier dysfunction, pro-inflammatory environment, neuroinflammation, excitotoxicity, oxidative stress, protein misfolding; gut–brain axis influences neuronal survival	Phenotypic heterogeneity; causal relationship between microbiota and neurodegeneration unclear; diet and antibiotics effects underexplored	Pro-, pre-, and postbiotics (butyrate); FMT; antioxidant/polyphenol-rich diets; ketogenic diets; nicotinamide modulation
IIMs (DM, PM, NAM)	Autoimmune	Reduced diversity; ↑ prevotellaceae, Streptococcus; ↓ butyrate-producers (Pseudobutyrivibrio, lachnospiraceae, Roseburia, Blautia, Faecalibacterium); subtype-specific dysbiosis	Dysbiosis contributes to immune dysregulation and systemic inflammation; SCFA-producing taxa influence amino acid metabolism and autoantibody levels	Small cohorts; heterogeneity across subtypes; causality vs. consequence unclear	Pro- and prebiotics to restore SCFA-producing taxa and modulate immune response
SMA	Genetic	Similar overall diversity to controls; qualitative differences: ↑ Gordonibacter, Ruminiclostridium, Enorma, Lawsonella, Frisingicoccus, Anaerofilum; ↓ Catabacter, Howardella, Marine_Methylotrophic_Group_3, Lachnospiraceae_AC2044_group; altered lipid metabolites	Dysbiosis may affect host metabolism through microbiota–metabolite interactions; functional consequences on SMA progression unclear	Small cohorts; mechanistic links between altered metabolites and SMA pathophysiology not fully understood	No established interventions; potential for microbiome-targeted metabolic modulation
GSD	Genetic/Metabolic	↓ firmicutes, bacteroidetes, lachnospiraceae, ruminococcaceae, peptostreptococcaceae, Blautia, E. hallii group, Faecalibacterium; ↑ actinobacteria, proteobacteria, bifidobacteriaceae, lactobacillaceae, veillonellaceae; A. muciniphila ↑; SCFA ↓, primary bile acids ↑	Dysbiosis alters metabolite profiles, reduces SCFA-mediated benefits, may contribute to GI and metabolic complications	Human data limited; small cohorts; causality vs. correlation unclear	Probiotics may reduce GI symptoms and improve quality of life; microbiota-targeted adjunctive therapies
CIDP	Autoimmune	↑ enterobacteriaceae, selenomonadaceae, acidaminococcaceae; ↓ bacteroidaceae, veillonellaceae, tannerellaceae, rikenellaceae; ↑ K. pneumoniae, E. coli, M. funiformis, P. faecium; ↓ Bacteroides spp.; ↑ bile acids and arachidonic acid	Shift from protective commensals to potentially pathogenic taxa; dysbiosis and metabolite alterations promote systemic inflammation and immunemediated neuropathy	Small cohorts; causal relationship between dysbiosis, metabolites, and pathophysiology not established	Potential for microbiome-targeted therapies to restore protective taxa and reduce inflammation (pro- and prebiotics)

Abbreviations: ALS, amyotrophic lateral sclerosis; A. muciniphila, Akkermansia muciniphila; ALS, amyotrophic lateral sclerosis; BMAA, β-methylamino-L-alanine; CIDP, chronic inflammatory demyelinating polyneuropathy; DM, dermatomyositis; DMD, Duchenne muscular dystrophy; *E. coli* , *Escherichia coli* ; *E. hallii* , *Eubacterium hallii* ; F/B, firmicutes/bacteroidetes; FMT, fecal microbiota transplantation; FSHD, facioscapulohumeral dystrophy; GM, gut microbiota; GSD, glycogen storage diseases; HDAC, histone deacetylase; IIMs, idiopathic inflammatory myopathies; K. pneumoniae, Klebsiella pneumoniae; LPS, lipopolysaccharides; M. funiformis, Megamonas funiformis; MD1, myotonic dystrophy type 1; MG, myasthenia gravis; NAM, necrotizing autoimmune myopathy; NMJ, neuromuscular junction; *P. faecium* , *Phascolarctobacterium faecium* ; PM, polymyositis; PPAR, peroxisome proliferator-activated receptor; SCFA, short-chain fatty acids; SMA, spinal muscular atrophy; TLR, toll-like receptor.

Note: The table includes disease category (autoimmune, genetic, or neurodegenerative), key microbial changes, proposed pathogenic mechanisms, knowledge gaps, and potential therapeutic implications.

**Table 2:** Functional consequences of gut microbiota dysbiosis in neuromuscular diseases

Disease	Key mechanistic effects of dysbiosis	Potential impact on disease
MG	↑ systemic inflammation, impaired Treg function, altered metabolites	Exacerbated autoimmunity at NMJ, muscle weakness, cognitive/psychiatric comorbidities
DMD	↑ intestinal permeability, ↑ systemic inflammation, altered lipid metabolism	Muscle inflammation, impaired function, disrupted gut–muscle axis
MD1	↑ systemic inflammation, altered SCFA levels, gut barrier dysfunction	Gastrointestinal symptoms, muscle weakness, altered pain perception
FSHD	↑ intestinal permeability, altered SCFA metabolism, HDAC modulation	Potential DUX4 overexpression, muscle injury, systemic immune activation
ALS	↑ neuroinflammation, impaired gut–brain axis, reduced neuroprotective metabolites	Accelerated neurodegeneration, worsened motor function
IIMs	↑ systemic/muscle inflammation, altered host metabolism	Muscle inflammation, antibody-mediated damage, organ involvement (ILD)
SMA	Altered lipid metabolism, potential neuromuscular modulation	Metabolic imbalance, impact on muscle health
GSDs	↑ bile acids, ↓ SCFAs, disrupted microbial interactions	Metabolic dysfunction, gastrointestinal symptoms
CIDP	↑ systemic inflammation, altered bile acid and arachidonic acid metabolism	Peripheral nerve inflammation, demyelination

Abbreviations: ALS, amyotrophic lateral sclerosis; CIDP, chronic inflammatory demyelinating polyneuropathy; DMD, Duchenne muscular dystrophy; DUX4, double homeobox 4 FSHD, facioscapulohumeral dystrophy; GSD, glycogen storage diseases; HDAC, histone deacetylase; IIMs, idiopathic inflammatory myopathies; ILD, interstitial lung disease; MD1, myotonic dystrophy type 1; MG, myasthenia gravis; NMJ, neuromuscular junction; SCFA, short-chain fatty acids; SMA, spinal muscular atrophy; Treg, regulatory T cell.

Note: The table summarizes the main mechanistic effects of gut microbiota (GM) alterations and their potential impact on disease pathophysiology across different neuromuscular conditions.

The perspective adopted in this review is informed by several years of clinical experience in the management of NMDs, within multidisciplinary care settings that frequently involve collaboration with pulmonologists and gastroenterologists. Such interactions often highlight the systemic manifestations and complications associated with these conditions. Exposure to this multidisciplinary context has contributed to the growing interest in systemic factors, including GM, that may influence the pathophysiology and clinical expression of neuromuscular diseases.

## MYASTHENIA GRAVIS (MG)

As an autoimmune NMD, MG is characterized by antibody-mediated dysfunction of neuromuscular junction (NMJ) transmission. Despite the well-established role of autoantibodies against acetylcholine receptors (AChR) and other NMJ components, MG pathogenesis remains incompletely understood and likely multifactorial. Emerging evidence suggests that GM alterations may contribute to disease onset and progression.

Notably, MG shares with other autoimmune disorders a common microbiome signature, including a reduced Firmicutes/Bacteroidetes (F/B) ratio driven by the relative expansion of bacteroidetes. 5 This imbalance has been linked to a proinflammatory microbial profile that may promote Th17 polarization and systemic immune dysregulation. 5 In MG, a specific dysbiotic pattern has been identified, with a decrease in actinobacteria, several firmicutes taxa such as *Clostridium* , *Eubacterium* , and *Lactobacillus* , and verrucomicrobiota, accompanied by an increase in proteobacteria, bacteroidetes, and thermodesulfobacteriota. 5 6 These microbial shifts are consistent with a proinflammatory state that may contribute to immune activation at the NMJ.

Parallel alterations have been described in the oral microbiota (OM), where firmicutes, actinobacteria, and bacillota are enriched, while spirochaetota and proteobacteria are reduced. 5 6 A previous study showed that MG led to a decrease in *Barnesiella* , yielding evidence of a causality connection in both directions between GM and MG. 7 Alterations in fecal metabolomic profiles have also been implicated in MG. 8

Metabolomic analyses revealed distinct fecal metabolic signatures between healthy controls and MG patients, identifying around 30 metabolites that could discriminate between the two groups. 8 Among these, 16 were reduced and 14 were elevated in MG. 8 Functional clustering indicated that these metabolites were primarily involved in amino acid metabolism (e.g., leucine, valine, cysteinylglycine), microbial metabolism across diverse environments (e.g., xanthine, oxalic acid, catechol), and nucleotide metabolism (e.g., cytosine, adenine, xanthine). 8 Such metabolic shifts may contribute to MG pathogenesis by reflecting or driving GM dysregulation and altered host–microbe metabolic interactions. 8

At the oral level, bacteria such as *Streptococcus* , *Rothia* , *Lachnoanaerobaculum* , and *Oribacterium* are increased in MG patients compared with healthy individuals, whereas *Neisseria* , *Haemophilus* , and *Treponema* appear decreased. 5 6 The relationship between OM and MG is likely multifaceted. Alterations in OM may promote proinflammatory immune responses capable of influencing systemic autoimmunity. 5 6 For example, *Streptococcus* species can modulate peroxisome proliferator-activated receptor-γ (PPAR-γ) signaling, affecting the balance between regulatory T and Th17 cells, while *Rothia* antigens are known to stimulate lymphocyte activation and macrophage-driven TNF-α production, potentially leading to acetylcholine receptor damage or enhanced B-cell differentiation and antibody production. 5 6 Oral dysbiosis may also interfere with the biosynthesis of ansamycin and amino acids such as valine, leucine, isoleucine, and glutamine. 5 6 Increased ansamycin levels—a macrolide compound—could theoretically impair neuromuscular transmission and exacerbate MG symptoms, whereas altered amino acid metabolism may exert additional immunomodulatory effects that influence disease onset and progression. 5 6

Emerging evidence indicates that GM alterations affect Foxp3 ^+^ CD4 ^+^ regulatory T cells (Tregs), contributing to the immunopathogenesis of MG. 8 MG features elevated antibodies against acetylcholine receptors (AChR-Abs), linked to imbalances among Th1 and B cells, and Foxp3 ^+^ Tregs. 8 Patients show reduced Foxp3 ^+^ Treg frequency, suggesting impaired immune tolerance. 8 These are abundant in the colonic lamina propria, are shaped by microbiota-driven antigenic stimuli that promote differentiation of naïve CD4 ^+^ T cells into regulatory subsets. 8 Functionally, Tregs suppress autoreactive T cells and B-cell antibody production, maintaining self-tolerance and limiting MG progression. 8 It is currently hypothesized that the dysbiosis-associated loss of specific commensals may disrupt Treg induction, weakening mucosal regulation and fostering systemic autoimmunity in MG. 8

Beyond direct immune mechanisms, it has been suggested that cognitive decline and psychiatric comorbidities in MG could, at least in part, reflect a systemic proinflammatory state secondary to dysbiosis and disruption of the gut–brain axis. 5 However, distinguishing whether these microbial alterations are a cause or a consequence of MG remains challenging.

Patients with MG are often exposed to multiple pharmacological agents, including proton pump inhibitors, corticosteroids, pyridostigmine, and immunosuppressants, all of which can substantially modify microbiota composition. 5 6 Furthermore, disease-related changes in diet, physical activity, and general lifestyle may further exacerbate dysbiosis. 5 6 Preliminary data suggest that modulating the GM via probiotics, prebiotics, and synbiotics may improve symptoms and immune balance in MG. 5 6

Fecal microbiota transplantation (FMT) is another experimental approach showing potential in altering disease outcomes. 8 9 Preclinical and early clinical studies indicate that restoring microbial balance can reduce inflammation and enhance neuromuscular function, positioning the microbiome as a promising therapeutic target. 5 6 Additionally, specific signatures linked to microbial shifts in firmicutes, bacteroidetes, and actinobacteria may serve as biomarkers for early diagnosis, disease stratification, and personalized microbiota-based treatments in MG. 5 6

In summary, current evidence suggests that gut and oral microbiota alterations may contribute to immune dysregulation in MG, particularly through modulation of Treg/Th17 balance and metabolic pathways. However, most available data are observational and potentially confounded by treatment and lifestyle factors, and thus a causal relationship remains to be firmly established.

## DUCHENNE MUSCULAR DYSTROPHY

Duchenne muscular dystrophy (DMD) is a severe X-linked disorder caused by mutations in the DMD gene, leading to dystrophin deficiency and progressive degeneration of skeletal and cardiac muscles. 10 Loss of dystrophin compromises muscle membrane stability, resulting in repeated damage-repair cycles that initially cause pseudo-hypertrophy but ultimately lead to fibrosis and irreversible weakness. 10 The disease primarily affects proximal muscles and progressively involves respiratory and cardiac muscles, culminating in severe disability and cardiorespiratory failure. 10 Secondary mechanisms, including chronic inflammation, altered calcium homeostasis, oxidative stress, mitochondrial dysfunction, and impaired autophagy, further contribute to disease progression. 10

Gastrointestinal disturbances observed in DMD suggest that both the composition and activity of the GM may be altered in this condition, potentially influencing host metabolism, inflammation, and muscle pathology. 11 Several factors common among DMD patients—such as reduced physical activity, chronic glucocorticoid therapy, and frequent antibiotic use for respiratory infections—can contribute to gut dysbiosis, thereby worsening intestinal inflammation and impairing barrier integrity. 11 Malnutrition, which often accompanies disease progression, may further exacerbate microbial imbalance and gastrointestinal dysfunction. 11 Nonetheless, it remains uncertain whether gut dysbiosis in DMD represents a contributing mechanism or merely an epiphenomenon secondary to the disease process.

Studies in *mdx* mice, the experimental model of DMD, have provided important insights into these gut–muscle interactions. 10 11 These mice exhibit abnormal intestinal peristalsis and distinct histopathological alterations in the mucosa, including enlarged villi, thinning of the muscular and submucosal layers, and increased nitric oxide production—features consistent with chronic inflammation. 10 11 Additionally, *mdx* mice show reduced fecal output, reflecting impaired intestinal motility likely linked to dysfunction of the migrating motor complex. 10 11 Although dystrophin is known to be expressed in smooth muscle, the specific effects of its absence in intestinal smooth muscle remain poorly understood.

Microbiota profiling in *mdx* mice revealed profound shifts in GM composition compared with wild-type controls, with reduced microbial richness and significant alterations across the phyla actinobacteria, proteobacteria, tenericutes, and *Deferribacteres* . 10 11 12 Some taxa, including the deferribacteraceae family and *Mucispirillum* genus, were found exclusively in dystrophin-deficient mice, suggesting a microbiota signature specific to DMD. 10 11

Furthermore, the overrepresentation of lipopolysaccharide (LPS)-producing Gram-negative bacteria, such as *Bacteroides* and members of deferribacteres, was associated with impaired intestinal motility, increased permeability, and reduced expression of tight junction proteins (Tjp1, Tjp2), all contributing to enhanced translocation of bacterial components and low-grade systemic inflammation. 10 11 This disruption of the intestinal barrier appears to amplify inflammation within dystrophic skeletal muscle. The *mdx* mice display upregulation of inflammatory mediators such as IL-6, TNF-α, and MCP-1, alongside activation of Toll-like receptor 4 (TLR4) signaling and its downstream effectors, including Myd88, Angptl4, and Bcat2, particularly in the tibialis anterior muscle. 10 11 Parallel increases in intestinal and systemic proinflammatory molecules—such as TRAF6, PMSB8, PTX-3, and RelB—further support the notion of a gut–muscle inflammatory axis in DMD. 10 11

Lipidomic analyses of *mdx* mouse intestines also demonstrated accumulation of lysophosphatidylcholines (LysoPC), derived from phosphatidylcholine cleavage, which correlated with an abundance of the bacterial genus *Alistipes* . 10 11 These lipid metabolites are known to activate TLR-dependent pathways, promoting oxidative stress and the release of proinflammatory cytokines such as IL-1β, IL-6, and TNF-α, as well as lymphocyte and macrophage activation. 10 11 Other studies have reported an increased abundance of *Alistipes* and *Prevotella* in *mdx* mice, correlating with altered T-cell populations in the spleen and muscle tissue, including expanded effector and memory subsets. 10 11 12

Moreover, pathways implicated in inflammatory bowel diseases—such as the IL-33/ST2 axis and intestinal permeability regulation—are similarly disrupted in DMD, underscoring shared mechanisms of chronic inflammation and barrier dysfunction. 10 These findings highlight the GM's key role in DMD pathogenesis: dysbiosis and impaired intestinal integrity exacerbate inflammation, disrupt immune balance, and affect muscle metabolism and function. 10 Notably, DMD patients with intellectual disability show relative abundance of *Propionibacterium* and *Bifidobacterium* , along with a decrease in *Bulleidia* —microbes involved in producing short-chain fatty acids that influence neurological function via the gut–brain axis—suggesting a link between microbiota composition and cognitive impairment. 13 Therapeutic studies in *mdx* mice show that PPARδ agonists, especially combined with AMPK inhibitors, improve muscle performance and reduce creatine kinase (CK) and oxidative metabolism proteins. 12 Other strategies under investigation include FMT, choline pathway inhibition, polyamine synthesis suppression, and taurine metabolism modulation, all of which aim to restore metabolic balance and reduce inflammation to improve muscle function in dystrophin-deficient models. 12

In summary, GM alterations appear to contribute to inflammation, barrier dysfunction, and muscle pathology in DMD, primarily through a gut–muscle axis supported by preclinical evidence. However, the lack of robust human studies and the presence of multiple confounding factors limit the ability to establish a clear causal role in patients.

## MYOTONIC DYSTROPHY

Type 1 myotonic dystrophy (MD1) is an autosomal dominant NMD caused by CTG trinucleotide repeat expansion in the 3' untranslated region of the *DMPK* gene, leading to progressive muscle weakness, atrophy, and myotonia. 11 Expanded repeats generate toxic CUG-containing RNA transcripts that disrupt pre-mRNA splicing and result in widespread protein dysfunction. 11 Clinical presentation is highly variable and correlates with repeat length and age at onset, with multiple phenotypes ranging from congenital to late-onset forms. 11 Beyond skeletal muscle involvement, MD1 is a multisystem disorder frequently affecting the central nervous system, heart, eyes, and endocrine system, and is associated with cognitive impairment, cataracts, cardiac conduction abnormalities, and endocrine and reproductive dysfunction. 11

Gastrointestinal disturbances are common in MD1 and may involve the entire digestive tract, from the pharynx to the anal sphincter. 11 Patients often experience alternating diarrhea and constipation, abdominal pain, bloating, and cramps. 11 Reduced gastrointestinal motility may lead to delayed gastric emptying, prolonged intestinal transit, or even gastroparesis, while other complications, such as gastroesophageal reflux, fecal incontinence, and hepatobiliary dysfunction, are also reported. 11 The pathophysiology of these gastrointestinal manifestations is likely multifactorial. 11 Although some studies have suggested a correlation between CTG repeat length and symptoms such as abdominal pain and constipation, increasing evidence points to a potential role of intestinal dysbiosis in their development. 11

Recent findings indicate that MD1 patients show an altered Firmicutes/Bacteroidetes ratio, a change possibly linked to softer stools and systemic inflammation. 11 Elevated circulating levels of IL-6, a proinflammatory cytokine associated with muscle weakness and reduced functional capacity, have also been observed, suggesting that microbiota imbalance may contribute to inflammatory processes in MD1. 11 Furthermore, the intestinal microbial profile of MD1 patients shows a lower abundance of actinobacteria—including *Corynebacterium* , *Rothia* , and *DNF00809* —compared to healthy controls, alongside a higher relative abundance of *Clostridium* CAG-352, lachnospiraceae, and *Prevotella* , and a reduction of *Lactobacillus* . 11 14 The depletion of *Lactobacillus* , a genus known for its probiotic and protective effects on gut health, may further exacerbate gastrointestinal symptoms such as bloating, cramping, and diarrhea by facilitating the overgrowth of pathogenic bacteria. 11 14

Furthermore, recent analyses have revealed that patients with MD1 exhibit a higher relative abundance of bacteroidetes and *Fusobacterium* compared to healthy controls, suggesting a distinct microbial signature associated with the disease. 14 Interestingly, several bacterial families within the firmicutes phylum—such as rikenellaceae, christensenellaceae, dehalobacteriaceae, oscillospiraceae, mogibacteriaceae, ruminococcaceae, sutterellaceae, desulfovibrionaceae, and erysipelotrichaceae—have been correlated with reduced severity of extra-intestinal pain symptoms. 14 Notably, these taxa were found to be relatively enriched in stool samples from MD1 patients, which could indicate a potential compensatory or protective microbial response. 14

This pattern suggests that specific microbial populations might play a modulatory role in systemic inflammation and pain perception in MD1, highlighting the complex interplay between GM composition and neuromuscular symptomatology. Recent findings have shown that the expansion of CTG repeats markedly alters the GM composition in homozygous mouse models of MD1, particularly affecting the relative abundance of the patescibacteria and deferribacterota phyla. 15 These alterations offer important insights into how repeat-associated genetic changes may reshape intestinal microbial communities, contributing to both the gastrointestinal disturbances and broader pathogenic mechanisms observed in MD1. 15

In pediatric patients with MD1, the abundance of the bacterial genus *Faecalibacterium* has been found to be markedly reduced compared to their healthy siblings. 16 Although *Faecalibacterium* remains among the four most dominant genera in both groups, its relative abundance is significantly lower in affected individuals (SD: 4.9 ± 2.1%) than in controls (SD: 9.1 ± 4.4%). 16 Given the well-established antiinflammatory and gut-protective roles of *Faecalibacterium* , restoring its levels may offer a therapeutic strategy to improve gut function and relieve bowel symptoms in MD1 patients. 16 Probiotic supplementation and tailored diets could help reduce malnutrition and gastrointestinal issues in this population.

Overall, dysbiosis appears to be associated with the gastrointestinal manifestations of MD1 and may also modulate systemic inflammatory pathways. Nevertheless, the available data is still preliminary, and a causal relationship between microbiota changes and disease features has yet to be established.

## FACIOSCAPULOHUMERAL DYSTROPHY

Facioscapulohumeral muscular dystrophy (FSHD) is an inherited NMD characterized by slowly progressive, often asymmetrical weakness of facial, scapular, humeral, and distal lower limb muscles. 17 It is the third most common adult-onset muscular dystrophy and includes two molecular subtypes, FSHD1 (∼ 95% of cases) and FSHD2 (∼ 5%), which differ in their genetic cause, but are clinically indistinguishable and share a common pathogenic mechanism: aberrant activation of the double homeobox 4 ( *DUX4* ) gene in skeletal muscle. 17 Normally epigenetically silenced, *DUX4* encodes a transcription factor whose inappropriate expression induces oxidative stress, inflammation, apoptosis, and impaired myogenesis, leading to muscle degeneration. 17 In FSHD1, contraction of the D4Z4 repeat array on chromosome 4q35 results in chromatin relaxation and *DUX4* derepression. 17

Recent findings suggest that GM dysregulation may also contribute to FSHD pathophysiology. Patients display a distinct microbial composition, with an increase in taxa such as enterobacterales, veillonellales-selenomonadales, and the *Selenomonadaceae* , *Sutterellaceae* , and *Veillonellaceae* families—microorganisms commonly implicated in gut dysbiosis and inflammatory bowel disease. 18 Conversely, short-chain fatty acid (SCFA)-producing genera such as *Alistipes* , *Butyricimonas* , and several UCG species are significantly reduced, suggesting an imbalance in microbial metabolites that normally support intestinal and systemic homeostasis. 18 A further notable feature is the rise in *Pasteurellaceae* species among patients with severe FSHD. 18 These bacteria can produce repeats-in-toxins (RTXs) with cytolytic and hemolytic properties, which could potentially exacerbate muscle cell injury. 18

Consistent with a disrupted gut–muscle axis, metabolic profiling revealed a reduction in fecal SCFA levels alongside their elevated concentrations in circulation, supporting the hypothesis of increased intestinal permeability or “leaky gut”. 18 Among these metabolites, butyric acid was found to be markedly elevated in plasma. Since butyrate functions as a histone deacetylase (HDAC) inhibitor, this increase may have mechanistic relevance, as HDAC inhibition has been shown to enhance *DUX4* expression in FSHD myocytes, potentially amplifying disease activity. 18 Alterations were also observed in medium- and long-chain fatty acids (MCFAs and LCFAs), which were decreased in fecal samples but elevated in blood, further supporting intestinal barrier dysfunction and systemic translocation of gut-derived metabolites. 18

Moreover, FSHD patients exhibited significant correlations between D4Z4 methylation patterns and circulating or fecal fatty acid profiles. 18 In particular, lower fecal concentrations of isobutyric, isovaleric, and 2-methylbutyric acids—molecules known to modulate immune function—were associated with hypomethylation of the D4Z4 array and evidence of systemic immune activation. 18 A strong association between proximal D4Z4 hypomethylation and increased circulating SCFAs (acetic, propionic, butyric, and valeric acids) further supports a link between epigenetic deregulation and metabolic alterations. 18 Reduced *Butyricimonas* abundance, together with diminished fecal levels of its derived SCFAs, underscores the disruption of the commensal equilibrium that normally sustains muscle and gut health. 18

Interestingly, decreased erysipelotrichales taxa correlated with elevated plasma isobutyric acid, an activator of the AKT signaling pathway—which is known to modulate *DUX4* expression. 18 Pharmacological inhibition of PI3K/Akt/mTOR signaling suppresses *DUX4* transcripts, indicating a link between microbial metabolites, signaling pathways, and gene expression in FSHD. 18 Overall, gut dysbiosis, altered SCFA metabolism, intestinal permeability, and *DUX4* regulation form a complex network where microbiome factors may influence disease severity and progression. 19

In summary, GM alterations in FSHD appear to be closely linked to disrupted SCFA metabolism, increased intestinal permeability, and epigenetic regulation of DUX4 expression, suggesting a potential role of the microbiome in modulating disease severity. However, current evidence is still limited and largely associative, and further studies are needed to establish causality and clinical relevance.

## AMYOTROPHIC LATERAL SCLEROSIS

Amyotrophic lateral sclerosis (ALS) is a progressive neurodegenerative disease primarily affecting upper and lower motor neurons, with frequent involvement of extra-motor domains. 20 21 Motor neuron degeneration leads to progressive muscle weakness and atrophy, with respiratory failure as the main cause of death, typically within 2 to 5 years of onset. 20 21 Up to half of patients develop cognitive or behavioral impairment, reflecting overlap with frontotemporal pathology. 20 21 Despite growing molecular insights, ALS diagnosis remains challenging and often delayed, relying mainly on clinical and neurological assessment. 20 21 Marked heterogeneity in disease course and prognosis, influenced by genetic and potential environmental factors, highlights the need for earlier diagnosis and personalized therapeutic approaches. 20 21

Recent research has increasingly highlighted that ALS is not only a neurodegenerative disorder confined to the central nervous system, but also a systemic condition involving the gut–brain axis. 22 Multiple studies have demonstrated that this cohort presents a markedly altered GM composition compared with healthy individuals, suggesting that microbial dysbiosis may play a role in disease onset or progression. 22 23 24 25 Broadly, ALS-associated dysbiosis is characterized by a disruption of major bacterial phyla, including a decrease in firmicutes and bacteroidetes—both essential for maintaining intestinal health and metabolic stability—although some studies have reported conflicting findings, underlining the need for further investigation. 22 23 24 25

At the genus level, beneficial commensals such as *Blautia* , *Ruminococcus* , and *Prevotella* are typically reduced, while potentially pathogenic taxa, including *Escherichia (E.)* and *Klebsiella (K.)* , may be increased. 22 These microbial shifts are thought to contribute to a pro-inflammatory intestinal environment, impaired barrier function, and altered production of neuroactive metabolites, all of which may influence neurodegeneration. 22 In line with these findings, it has been reported that ALS patients exhibited a reduction in firmicutes and *Megamonas* , accompanied by an increase in bacteroidetes when compared with healthy controls. 26 Conversely, another investigation identified an elevated F/B ratio in a smaller ALS cohort, along with higher *Methanobrevibacter* levels and a decline in beneficial genera such as *Faecalibacterium* and *Bacteroides* . 26 Similarly, a comparative study involving ALS patients, their healthy relatives, and matched controls revealed distinct microbial signatures, including lower abundances of *Succinivibrionaceae* and *Lachnospiraceae* , and increased *Streptococcaceae* and ruminococcaceae, underscoring the heterogeneity of GM alterations in ALS. 23 24 25 26

A particularly relevant finding is the reduction in SCFA-producing bacteria, such as *Butyricimonas* and *Faecalibacterium* . These SCFAs—particularly butyrate—exert potent antiinflammatory and neuroprotective effects by promoting regulatory T-cell differentiation, reducing proinflammatory cytokines, and maintaining intestinal and blood–brain barrier integrity. 22 Moreover, butyrate supports neuronal survival through multiple mechanisms: inhibition of HDACs, enhanced expression of neurotrophic factors like brain-derived neurotrophic factor (BDNF), upregulation of antioxidant enzymes, such as superoxide dismutase (SOD) and catalase, and stimulation of autophagy for the removal of misfolded proteins. 22

Experimental studies in ALS animal models have shown that butyrate supplementation improves motor performance, prolongs survival, and limits motor neuron degeneration, reinforcing its therapeutic potential. 22 Conversely, reduced SCFA levels in ALS could weaken these protective mechanisms, exacerbating neuroinflammation and accelerating neurodegeneration. 22 Alongside beneficial metabolites, harmful bacterial products may also contribute to ALS pathology. The LPS released by Gram-negative bacteria are known to trigger systemic inflammation and have been detected at elevated levels in ALS patients. 22

Other microbial-derived molecules, such as sphingolipids, bile acids, and endocannabinoid-like compounds, could further disrupt host–microbiota homeostasis. 22 Of particular concern is β-methylamino-L-alanine (BMAA), a neurotoxin produced by cyanobacteria that has been implicated in ALS pathogenesis. 22 Exposure to BMAA—whether through contaminated food, water, or air—has been associated with increased ALS incidence in several geographic clusters, including Guam and Japan's Kii Peninsula. 22 Mechanistically, this neurotoxin induces excitotoxicity, oxidative stress, and protein misfolding by substituting serine residues during protein synthesis. 22 Intriguingly, some evidence suggests that gut dysbiosis might enhance endogenous BMAA production, linking microbial imbalance directly to neurotoxicity in ALS. 22

Phenotypic differences may further shape microbiota composition. Patients with bulbar-onset ALS, who experience early dysphagia, show more profound dysbiosis than those with spinal-onset forms. 22 Difficulty swallowing reduces dietary fiber intake, leading to malnutrition and decreased microbial diversity. 22 This promotes the overgrowth of pathogenic species such as *E. coli* and *K. pneumoniae* , which produce molecules capable of entering circulation and exacerbating systemic inflammation. 22 Moreover, enteral feeding—often required in advanced stages—alters nutrient digestion and absorption, further disturbing microbial equilibrium and reducing SCFA synthesis. 22

Another factor influencing microbiota health in ALS is antibiotic exposure. 22 Antibiotic use in ALS patients reduces microbial diversity, promotes resistant pathogens, and worsens inflammation, disrupting neuroprotective pathways. 22 Diet also influences microbiota health: high-protein, low-fiber diets can suppress SCFA production and induce dysbiosis, while fiber-rich or prebiotic diets may restore microbial balance, improve gut barrier function, and reduce neuroinflammation. 22 Together, these factors contribute to metabolic and inflammatory cascades that increase neuronal vulnerability in ALS. 22 23 24 25

Microbiome modulation through probiotics, prebiotics, diet, and postbiotics like butyrate offers a promising, yet underexplored, approach to improve ALS outcomes. 26 Nutritional strategies rich in antioxidants and polyphenols can favor beneficial gut bacteria, reduce inflammation, and support neuroprotection by modulating metabolites and inhibiting protein aggregation. 26 High-fat diets, such as ketogenic regimens, may also benefit neuronal energy supply and microbiota composition. 26 While generally safe, microbial therapies like probiotics and FMT carry risks—especially in immunocompromised patients—necessitating careful risk–benefit assessment. 26 Notably, *Akkermansia muciniphila (A. muciniphila)* has been linked to increased nicotinamide, which promotes neuronal survival in ALS models. 26 27

Overall, accumulating evidence supports a role of the GM in influencing ALS pathophysiology through immune, metabolic, and neurotoxic pathways. Nevertheless, the variability across studies and the influence of confounding factors limit definitive conclusions, highlighting the need for well-controlled clinical investigations.

## IDIOPATHIC INFLAMMATORY MYOPATHIES

Idiopathic inflammatory myopathies (IIM) comprise a heterogeneous group of disorders, including dermatomyositis (DM), polymyositis (PM), overlap myositis, inclusion body myositis (IBM), and necrotising autoimmune myopathy (NAM). 28 Most IIMs present with proximal muscle weakness and elevated CK levels, whereas IBM typically affects the quadriceps and finger flexors with normal or mildly increased CK. 28

Advances in serological and imaging techniques are reshaping diagnosis and reducing reliance on muscle biopsy, while expanding therapeutic options—such as intravenous immunoglobulin and rituximab—offer benefit in refractory cases. 28 In recent years, growing evidence has highlighted the GM as a key contributor to the pathophysiology of IIMs. 29

Several studies have demonstrated that patients with IIM exhibit a markedly reduced microbial diversity compared to healthy controls. 29 30 In particular, members of the prevotellaceae family—often associated with inflammatory processes—were found to be increased, whereas beneficial butyrate-producing bacteria such as *Pseudobutyrivibrio* , lachnospiraceae, *Roseburia* , and *Blautia* were significantly reduced. 29 30 Interestingly, the abundance of butyrate-producing taxa like lachnospiraceae and *Pseudobutyrivibrio* correlated positively with elevated levels of amino acids such as L-leucine and asparagine, suggesting a link between microbial composition and host metabolism. 29 30 In juvenile DM, a distinct dysbiosis has also been reported, characterized by a reduction in *Faecalibacterium* within the gut and alterations in the OM, notably an increased presence of *Streptococcus* . 31

Similarly, patients with NAM show a unique microbial signature, with decreased abundances of bacteroides, *Roseburia* , and *Coprococcus* , and elevated levels of *Lactobacillus* and *Streptococcus* . 32 These compositional changes appear to have immunological relevance: *Lactobacillus* abundance correlates positively with antisignal recognition particle (SRP) and anti-Ro52 antibodies, as well as with erythrocyte sedimentation rate (ESR); *Streptococcus* levels correlate with anti-HMGCR antibodies and C-reactive protein (CRP). 32

Conversely, *Roseburia* abundance is inversely associated with markers of muscle and systemic inflammation such as myoglobin (MYO), cardiac troponin T (cTnT), ESR, CRP, and the occurrence of interstitial lung disease (ILD). 32 Negative correlations were also observed between *Bacteroides* and both ESR and CRP, and between *Coprococcus* and ESR. 32 Moreover, causal relationships have been proposed between specific bacterial genera and distinct IIM subtypes—for instance, ruminococcaceae, *Sutterella* , and *Anaerotruncus* with DM, and *Alloprevotella* , *Ruminococcaceae UCG003* , and *Dialister* with PM. 33 Altogether, these findings suggest gut dysbiosis reflects and may drive immune dysregulation and inflammation in IIMs.

In summary, GM alterations in IIMs are characterized by reduced microbial diversity, depletion of butyrate-producing taxa, and enrichment of proinflammatory bacteria, suggesting a potential role in modulating immune activation and disease activity. However, most evidence is based on cross-sectional and associative studies, and causal relationships remain to be established.

## SPINAL MUSCULAR ATROPHY

Spinal muscular atrophy (SMA) is an autosomal recessive neurodegenerative disorder caused by biallelic mutations in the survival motor neuron 1 ( *SMN1* ) gene, leading to progressive loss of alpha motor neurons and subsequent muscle weakness and atrophy. 34 Disease severity is highly variable and largely influenced by the copy number of the *SMN2* gene, which partially compensates for *SMN1* loss and generally correlates inversely with clinical severity. 34 Prior to the introduction of SMN-enhancing disease-modifying therapies (DMTs), SMA was classified into distinct types based on age at onset and motor milestone achievement. 34

Recent research has explored the GM of patients with SMA, revealing no significant differences in overall microbial diversity compared with matched healthy controls. 35 However, the microbial composition showed clear qualitative differences. 35 While both groups shared bacteroidetes and firmicutes as dominant phyla, specific bacterial genera varied notably. 35 The F/B ratio—often associated with intestinal inflammation—did not differ significantly between the two groups. 35 Nevertheless, the SMA cohort showed a higher relative abundance of *Gordonibacter* , *Ruminiclostridium* , and *Enorma* compared to controls, indicating a distinct microbial profile. 35 Additional genera enriched in SMA included *Lawsonella* , *Frisingicoccus* , and *Anaerofilum* , whereas *Catabacter* , *Howardella* , *Marine_Methylotrophic_Group_3* , and *Lachnospiraceae_AC2044_group* were reduced. 35 Metabolomic analyses identified 56 lipid metabolites differing between SMA patients and controls, with correlations to specific bacterial taxa, suggesting a link between GM and metabolic changes in SMA. 35

In conclusion, SMA is characterized by subtle changes in GM composition and associated lipid metabolism, while overall diversity remains largely preserved. Further studies are required to determine whether these alterations have functional or clinical significance.

## GLYCOGEN STORAGE DISEASES

Glycogen storage diseases (GSDs) are inherited metabolic disorders caused by defects in enzymes or transport proteins involved in glycogen synthesis or degradation, leading to impaired glycogen utilization and abnormal intracellular accumulation. 36 With an incidence of 1 in 20,000 to 1 in 43,000 live births, over 20 types and subtypes have been identified, classified by enzyme deficiency and tissue involvement. 36 While liver and skeletal muscles are most commonly affected due to glycogen storage, other organs such as heart, kidneys, and brain may also be involved, resulting in multisystem manifestations. 36

In a previous study investigating the GM and microbial metabolism in children with hepatic GSD, researchers observed a marked alteration in microbial composition. 37 Specifically, the relative abundance of the phyla firmicutes and bacteroidetes—along with the families lachnospiraceae, ruminococcaceae, and peptostreptococcaceae, and the genera *Blautia* , *Eubacterium hallii (E. hallii)* group, and *Faecalibacterium* —was significantly decreased. 37 Conversely, increases were reported in actinobacteria and proteobacteria phyla, bifidobacteriaceae, lactobacillaceae, and veillonellaceae families, as well as in *Lactobacillus* , *Enterobacter* , and *Veillonella* genera. 37 Metabolically, these microbial shifts were associated with elevated levels of primary bile acids and reduced concentrations of SCFAs. 37

In another study, 38 patients with GSD exhibited higher relative abundances of enterobacteriaceae and veillonellaceae families, while beneficial genera such as *Faecalibacterium* and *Oscillospira* were markedly reduced. Although fecal SCFA analysis revealed increased levels of acetate and propionate, their positive physiological effects appeared limited, likely due to disrupted bacterial interactions. 38

More recently, further evidence confirmed a significant reduction in firmicutes and an increase in actinobacteria in GSD patients compared to healthy controls. 39 At the genus level, *Akkermansia* , *Pseudoalteromonas* , *Uruburella* , and *Castellaniella* were predominant in GSD patients, whereas *Faecalibacterium* , *Bacteroides* , *Gemmiger* , and *Parabacteroides* prevailed in the control group. 39 Species-level analysis revealed a notable decrease in *Faecalibacterium prausnitzii (F. prausnitzii)* and an enrichment of *A. muciniphila* in children with GSD. 39

Probiotic supplementation shows therapeutic potential. 40 In a GSD type Ia case with inflammatory bowel disease, probiotics reduced intestinal symptoms and improved quality of life, indicating a promising adjunctive role for microbiota-targeted therapies. 40

In summary, patients with GSD exhibit consistent alterations in GM composition, characterized by depletion of SCFA-producing bacteria and enrichment of taxa associated with dysbiosis, together with relevant metabolic changes involving bile acids and SCFAs. However, the current evidence is still limited and largely based on small cohorts, and the functional implications of these alterations require further investigation.

## CHRONIC INFLAMMATORY DEMYELINATING POLYRADICULONEUROPATHY

Chronic inflammatory demyelinating polyradiculoneuropathy (CIDP) is a rare, immune-mediated neuropathy characterized by peripheral nerve demyelination, causing progressive muscle weakness and sensory deficits. 41 It typically follows a gradual or relapsing course lasting over 8 weeks, with electrophysiological evidence of demyelination and responsiveness to immunomodulatory therapies. 41 Pathogenesis involves both humoral and cellular immune mechanisms, including autoantibodies against gangliosides and autoreactive T cells targeting myelin, reflecting multiple autoimmune pathways. 41

In a comparative analysis of GM, patients with CIDP displayed a clear dysbiotic pattern relative to non-CIDP controls. 42 At higher taxonomic levels, members of the enterobacteriaceae, selenomonadaceae, and acidaminococcaceae families were relatively enriched in CIDP, whereas bacteroidaceae, veillonellaceae, tannerellaceae, and rikenellaceae were depleted. 42 This imbalance was reflected at the genus and species levels: CIDP stool samples showed increased representation of potentially pathogenic taxa such as *Klebsiella* (notably *K. pneumoniae* ), *Escherichia coli (E. coli)* , *Megamonas funiformis (M. funiformis)* , and *Phascolarctobacterium faecium (P. faecium)* , while commensal *Bacteroides* spp. (including *B. uniformis* , *P. dorei* and *P. vulgatus* ) were reduced. 42 Overall, these findings point to a shift from indigenous, presumably protective bacteria toward taxa with pathogenic potential. 42 Metabolomic profiling showed elevated bile acids and arachidonic acid in CIDP, likely reflecting altered microbiota and contributing to systemic inflammation and disease mechanisms. 42

Overall, CIDP is associated with a distinct GM signature characterized by enrichment of potentially pro-inflammatory taxa and depletion of commensal bacteroidetes, alongside metabolomic alterations involving bile acids and arachidonic acid. However, the current evidence is based on limited comparative studies, and the causal contribution of these changes to disease pathogenesis remains to be established.

## STRENGTHS AND LIMITATIONS

This review provides a comprehensive synthesis of current evidence on the GM in NMDs, systematically summarizing available literature and integrating emerging therapeutic perspectives. Beyond describing disease-specific microbial alterations, it proposes an original interpretative framework in which the GM is considered a relevant pathophysiological factor, as well as a potential biomarker and therapeutic target.

Nevertheless, several limitations should be acknowledged. The available studies are highly heterogeneous in design and methodology, reflecting the uneven development of research across different NMDs. While conditions such as MG, ALS, and MD1 are supported by relatively consistent evidence, data on rarer diseases remain scarce and preliminary. Additionally, most studies rely on small cohorts and are susceptible to confounding factors, including diet, treatments, disease stage, and comorbidities, thereby limiting the generalizability of current findings.

## FUTURE PERSPECTIVES AND CONCLUSIONS

Considering the role of the GM is pivotal for achieving a more integrated understanding of the pathogenesis of NMDs. Characterizing its composition and alterations may provide novel insights into disease mechanisms and reveal therapeutic targets beyond the nervous system.

Modulation of GM and dysbiosis correction could represent promising therapeutic strategies, particularly in conditions lacking disease-modifying treatments. Moreover, the GM may function as a biomarker for diagnosis and follow-up, reflecting disease progression and severity and supporting patient stratification and personalized management. Given its dynamic nature and susceptibility to pharmacological influences such as antibiotics, steroids, and immunosuppressants, GM assessment is also relevant when designing therapeutic approaches.

Importantly, while dysbiosis generally contributes to disease progression through mechanisms such as systemic inflammation, altered metabolite production, and impaired gut barrier function, the specific effects differ among neuromuscular diseases. Autoimmune conditions (e.g., MG, IIMs) are mainly affected via immune dysregulation, whereas genetic disorders (e.g., DMD, SMA) are influenced by metabolic and gut–muscle interactions, highlighting the need for disease-specific microbiota-targeted strategies.

Overall, investigating the GM offers a broader, systemic perspective on NMDs, while further studies are required to clarify causal relationships and the therapeutic potential of microbiota-based interventions.
